# The ‘UPIC’ cohort: a nationwide prospective study of mental health among adolescents and young adults in Sweden – a study protocol

**DOI:** 10.1136/bmjopen-2026-120432

**Published:** 2026-09-21

**Authors:** Emma Hovén, Andreas Frick, Alkistis Skalkidou, Fotios C Papadopoulos, Lisa Ekselius, Malin Gingnell, Rose-Marie Karlsson, Karin C Brocki, Hanna Ljungvall, Emma Ahlqvist Lindqvist, Rakel Eklund, Jonas Bergquist, Lina Eklund, Ellen Nilses, Stina Hallsén, Pernilla Åsenlöf

**Affiliations:** 1 Department of Women’s and Children’s Health, Physiotherapy and Behavioral Medicine, Uppsala University, Uppsala, Sweden; 2 Department of Psychology, Uppsala University, Uppsala, Sweden; 3 Department of Medical Sciences, Experimental Cognitive and Affective Neuroscience Lab, Uppsala University, Uppsala, Sweden; 4 Department of Women’s and Children’s Health, Obstetric and Reproductive Health Research, Uppsala University, Uppsala, Sweden; 5 WOMHER - Uppsala University Centre for Women’s Mental Health, Uppsala University, Uppsala, Sweden; 6 Department of Medical Sciences, Clinical Psychiatry, Uppsala University, Uppsala, Sweden; 7 Department of Social Work, Uppsala University, Uppsala, Sweden; 8 Department of History of Science and Ideas, Centre for Medical Humanities, Uppsala University, Uppsala, Sweden; 9 Department of Women’s and Children’s Health, Medical Psychology and Care Science, Uppsala University, Uppsala, Sweden; 10 Department of Chemistry for Life Sciences, Analytical Chemistry, Uppsala University, Uppsala, Sweden; 11 Department of Informatics and Media, Uppsala University, Uppsala, Sweden; 12 Target ‑Group Representative and Research Partner, Uppsala University, Uppsala, Sweden; 13 Department of Education, Uppsala University, Uppsala, Sweden

**Keywords:** MENTAL HEALTH, Adolescent, Depression & mood disorders, Anxiety disorders, PREVENTIVE MEDICINE

## Abstract

**Abstract:**

**Introduction:**

Rates of adolescents and young adults (AYAs) reporting poor mental health have increased, with symptoms of anxiety and depression being most common. Yet, the processes underlying these trends remain unclear. This cohort study aims to establish a comprehensive dataset combining data from multiple sources (surveys, behavioural tests, registers and smartphone sensors) to assess mental health, including mental ill-health and well-being. The dataset will enable analyses of prevalence, risk and protective factors and predictive modelling of mental health trajectories. The study also evaluates the feasibility of such longitudinal data collection among AYAs.

**Methods and analysis:**

This study recruits 2000 AYAs aged 15–29 years from the general population in Sweden using an initial random sample invited by post, followed by recruitment through digital channels. Participants are followed for 2 years under one of three protocols: annual assessments, bi-annual assessments and annual assessments complemented by repeated ecological momentary assessments. Across protocols, data are collected through surveys using a study-specific mobile app and digital behavioural tests. Demographic and clinical information, including psychiatric diagnoses and prescribed medications, are obtained through national health registers. Smartphone sensor data are collected for insights into phone usage, sleep patterns and mobility. Blood samples are collected from a subset via home-based testing to enable biomarker analysis. When applicable, machine learning, including deep learning techniques, is applied to develop predictive algorithms for mental health outcomes, with the potential to inform early identification and prevention. Feasibility outcomes include recruitment and retention rates and the acceptability of study procedures and measures, assessed to inform future studies.

**Ethics and dissemination:**

Representatives from the target population have contributed to the study’s development. The study complies with General Data Protection Regulation (GDPR) and has been approved by the Swedish Ethical Review Authority. Findings will be disseminated through scientific publications, conferences and to the public and relevant organisations.

**STRENGTHS AND LIMITATIONS OF THIS STUDY**

- A two-stage sampling strategy, starting with a random population-based sample followed by digital channel recruitment and follow-up aimed at under-represented groups, to mitigate selection bias and enhance representativeness of the sample.
- Prospective collection of data from multiple sources with repeated assessments of mental ill-health and well-being over 2 years.
- Integration of data from multiple sources enables detailed characterisation of mental ill-health and well-being, and their prediction.
- Randomisation to three distinct data assessment protocols allows evaluation of protocol-specific effectiveness and feasibility.
- Minimal participant-researcher interaction and ambitious protocol with a potentially heavy workload on respondents, increases risk of dropout and potential attrition bias, which will be evaluated as part of the feasibility analysis.

## Introduction

 Mental disorders are a leading cause of disability worldwide, imposing a substantial burden on individuals and societies.^1 2^ Adolescents and young adults (AYAs) aged 15–29 years constitute a particularly high-risk group compared with other age cohorts,^3 4^ with rising rates of mental ill-health reported both in Sweden and globally.^5 6^ Understanding mental health during this developmental period is critical, as adolescence and young adulthood involve profound biological, emotional, cognitive and social changes that shape identity formation, decision-making and relationships.^7–10^ Most mental disorders debut during this period, with a peak onset around age 15 years and a median onset between 18 and 20 years.^11–13^ According to the WHO, one in seven adolescents experiences a mental disorder,^14^ and recent European data indicate that one in four females aged 15–19 years lives with a mental health condition,^2^ highlighting that young women are disproportionately affected.^2–4^ Despite these high, and in some groups rising rates, the underlying drivers remain poorly understood. Mental disorders are the leading cause of years lost because of disability in young people aged 10–24 years,^15^ with mood and anxiety disorders being the most common.^16^ Moreover, suicide remains the leading cause of death among AYAs.^2^ Mental disorders in young age, particularly depression, are associated with increased risks of adult psychopathology, including anxiety, substance use disorder and suicidality.^17–20^ Thus, deeper insight into the determinants, trajectories and contexts of these trends is essential for developing effective strategies that promote mental well-being and prevent mental ill-health.

Mental health is a broad, multidimensional construct that includes both mental well-being and mental ill-health. Mental ill-health is commonly conceptualised along a continuum ranging from common symptoms of distress—such as worry, stress and emotional strain—to severe mental disorders, including major depressive disorder and psychosis.^21^ This conceptualisation emphasises that mental ill-health is not limited to diagnosable disorders, reflecting a spectrum of experiences marked by varying degrees of distress and functional impairment. At the same time, mental health is increasingly understood within a dual-factor framework, which conceptualises mental ill-health and mental well-being as related but distinct constructs.^22^ This perspective recognises that mental ill-health may cooccur with high levels of well-being and life satisfaction, while the absence of mental ill-health does not necessarily correspond to high levels of well-being. A growing body of research, including studies conducted among adolescents in Sweden, supports the dual-factor model of mental health.^23–25^ This framework underscores the importance of considering both mental ill-health and mental well-being when conceptualising, operationalising and measuring mental health.

Existing research on mental health among AYAs is limited by inconsistent measurement approaches, reliance on data from one source and predominantly use of cross-sectional designs, all of which limit the ability to capture the complexity and temporal dynamics of mental health. In Sweden, research examining mental health and its determinants among young people has increased substantially in recent years.^26–30^ However, there remains a need for studies that provide comprehensive estimates of common mental health conditions including but not limited to depression and anxiety and that integrate multiple dimensions of mental health and data sources within a longitudinal framework.^21^ Globally, there is increasing recognition of the need for multidimensional assessment approaches to better capture the complexity and heterogeneity of mental health and its trajectories.^31^ In response, researchers and health systems are increasingly adopting principles of precision medicine and individualised care to improve prevention and intervention efforts.^32 33^ However, most existing datasets remain insufficiently equipped to capture the complexity and heterogeneity of mental health phenotypes,^18^ prompting growing investment in large-scale, prospective population cohorts designed to collect multidimensional data from multiple sources.

Several risk factors for anxiety and depression among AYAs have been identified.^34 35^ Among important determinants, health behaviours, such as physical activity, sleep, alcohol use, smoking, cannabis use, diet and use of social media, are known to be associated with mental health outcomes.^36–39^ These health behaviours represent important modifiable targets for prevention, yet mental health may also influence an individual’s ability to engage in healthy behaviours, suggesting reciprocal associations that warrant further investigation. To fully understand these dynamics, research must move beyond isolated risk factors and adopt integrated datasets that combine biological, psychological, behavioural and social dimensions. Accordingly, the present study adopts a biopsychosocial framework,^40^ with the aim of capturing the interacting psychological, social, behavioural and biological dimensions of mental health. Such approaches are essential for capturing the complexity of mental health trajectories and for developing predictive models that can inform timely, personalised prevention and intervention strategies. Furthermore, a comprehensive dataset provides opportunities for researchers from different disciplines to investigate emerging research questions, generate and test hypotheses and apply diverse theoretical models.

The widespread use of smartphones, together with advances in technology and computational methods, offers opportunities to monitor mental health over time. Digital phenotyping, defined as the moment-by-moment quantification of the individual-level human phenotype using data from digital devices,^41 42^ includes active data, which requires user engagement (eg, surveys), and passive data, which is automatically collected through for example smartphone sensors and activity logs.^43^ These passive data complement active data of self-reported measures, enabling a multidimensional assessment of health with indicators of mobility, sleep patterns and digital social activity. Digital phenotyping data hold potential for the multidimensional assessment of health,^44^ and detecting, characterising and predicting mood disorders,^45 46^ anxiety^47^ and depressive symptoms.^48 49^

Ecological momentary assessment (EMA) represents an active component of digital phenotyping, complementing passive sensor data by capturing real-time self-reports of symptoms and experiences in naturalistic settings.^33 50^ This approach typically employs brief surveys administered once or multiple times per day to assess momentary states and contextual influences. Advances in smartphone technology have made EMA both feasible and well-suited to capture experiences in real-world context. Smartphone use is pervasive among today’s youth in Sweden, with recent statistics from 2024 showing that all AYAs in the age group 15–24 years had access to a smartphone, facilitating the uptake of such technologies. Compared with traditional self-report instruments that require a respondent to describe their experience or symptoms over a certain time period, EMA minimises recall bias and enhances ecological validity by collecting data in real time.^33^ The ability of EMA to capture dynamic, real-world processes has led to its increasing use in mental health research. However, the benefits may be compromised by challenges related to participant adherence and compliance. Although adherence varies across studies and EMA protocols, a recent systematic review and meta-analysis found moderate to high enrolment, low dropout and compliance levels approaching the commonly used 80% benchmark in studies of children and adolescents.^51^ These findings support the feasibility of EMA in youth populations while highlighting the importance of monitoring adherence throughout data collection.

Underlying behaviours and decisions in real life, as captured by the passive behavioural data, survey responses and registry data, are various psychological processes. These processes can be probed using remote psychological tests and computational models to link cognition and behaviour in well-controlled experimental contexts to real-life settings, providing a seldom used, but potentially highly important linkage between mental health and behaviour. In the context of mental health, avoidance of discomfort, decision-making under uncertainty and executive functions are of special interest.^52–54^ In addition to psychological, behavioural and social factors, biological processes may provide important information for understanding mental health trajectories and identifying individuals at increased risk of future mental ill-health. Integrating biological data may improve both the characterisation and prediction of mental health outcomes. For instance, metabolomics biomarkers have gained interest for their ability to screen new and unsuspected pathways involved in psychiatric disorders.^55^

To advance the field, it is essential to extend the understanding of the dynamics of how multiple factors impact mental health among AYAs, how mental health-related parameters fluctuate over time and which factors contribute to resilience or vulnerability. Such knowledge is critical for predicting the development of mental ill-health and enabling timely, personalised preventive interventions. Tracking individuals across critical developmental periods, using innovative methods such as digital phenotyping and remote psychological testing, offers unprecedented opportunities to characterise mental health trajectories with high precision. Nevertheless, to our knowledge, no previous study has implemented an approach that combines digital phenotyping with biological and behavioural measures to comprehensively examine mental health among AYAs in the Swedish general population.

### Objectives

The study aims to advance the understanding of mental health, including both mental ill-health and well-being, among AYAs by leveraging large-scale data, collected in real-world settings from multiple sources. The overarching goal is to characterise mental health outcomes and trajectories over time, in order to improve identification of AYAs at risk for mental ill-health and to inform early support and precision mental healthcare at critical developmental periods. A key objective is to evaluate the feasibility and acceptability of large-scale longitudinal data collection, including real-time digital phenotyping and EMA.

## Methods and analysis

This study is conducted as part of the interdisciplinary research programme ‘UPIC – Young People’s Mental Health in Focus’. The programme was initiated in response to reports of deteriorating mental health among AYAs, with the aim of advancing the current state of knowledge by developing a comprehensive dataset that combines established and innovative approaches to data collection and analysis. The study is guided by a biopsychosocial framework based on the premise that mental health reflects complex interactions among psychological, social, behavioural and biological factors. Accordingly, researchers from psychology, epidemiology, behavioural medicine, social sciences and related disciplines collaborated in the design of the study. Consistent with the biopsychosocial framework, measures were selected to capture psychological, social, behavioural and biological dimensions of mental health and its determinants, drawing on existing literature and iterative interdisciplinary discussions within the UPIC programme.

### Study design

This study is a prospective, population-based cohort including 2000 AYAs aged 15–29 years at study start recruited from the general population in Sweden. The sample size of 2000 participants is based on statistical precision, analytic requirements and feasibility considerations. Participants are followed for 2 years with repeated assessments of mental ill-health and well-being. The 2-year follow-up was chosen to evaluate the feasibility of this comprehensive multi-source data collection approach while examining mental health trajectories during a critical developmental period and generating hypotheses to inform future follow-up studies and further cohort development. To compare the feasibility and effectiveness of alternative data collection strategies for characterising and predicting mental health outcomes, participants are randomly assigned to one of three data collection protocols that vary in intensity (ie, how frequently participants are asked to provide data) and in the types of data sources included within each protocol.

### Cohort description

A two-stage sampling strategy was applied, starting with a random population-based sample followed by recruitment through digital channels and follow-up aimed at under-represented groups, to enhance representativeness of the general population sample. In the first stage, a random, age- and sex-stratified selection (n=40 000) of women and men aged 15–29 years was drawn from the Swedish population register (Navet).^56^ From this sample, postal invitations were sent to two random subsamples stratified by age and sex, each comprising 9000 individuals. The subsamples were invited sequentially because the recruitment rate was uncertain at the outset. This stepwise invitation procedure allowed controlled monitoring of recruitment rates and informed adjustments to the recruitment strategy, while avoiding unnecessary postal costs and over-recruitment. The invitation clearly stated that participation was open to all individuals aged 15–29 years, regardless of mental health status. Interested individuals were encouraged to use the QR code provided to download the UPIC app, which initiated the recruitment process. In the second stage, recruitment was supplemented by a digital open advertisement campaign. Advertisements were published on social media platforms including Facebook, Instagram and TikTok, targeting individuals aged 15–29 years, again regardless of previous or current mental health status. Following an initial skew towards female participants, the campaign was adjusted to increase participation of men.

Participants registered for the study via the study app, with identity verification through BankID (the most common secure online identification method in Sweden) or electronic signature for those without BankID. Recruitment began in August 2024 and continued until the target of 2000 participants at baseline. While the recruitment to the baseline assessment is now (March 2026) close to being completed (n=1957), data collection at follow-ups is ongoing up to 2 years after enrolment date.

### Data collection

Data are collected over a 2-year period using standardised assessments from multiple sources (figure 1). The UPIC smartphone app was developed specifically for this study, building on the features and insights from the established Mom2B app, which has been successfully used for digital phenotyping and detection of perinatal mental health outcomes.^57 58^ Both apps are built using the Beiwe research platform (www.beiwe.org) developed by the Harvard School of Public Health. The UPIC app serves as the primary platform for data collection and digital phenotyping, collecting both active (survey, behavioural tests) and passive data (sensor and log). Only data for which participants have provided consent are displayed in the app from the time of registration, and participants can modify their consent preferences at any point during the study regarding which data to be collected. Notifications are sent via the app and by email when new assessments by survey and behavioural tests are available. Participants can customise notification settings, including the preferred time of day to receive notifications.

**Figure 1:**
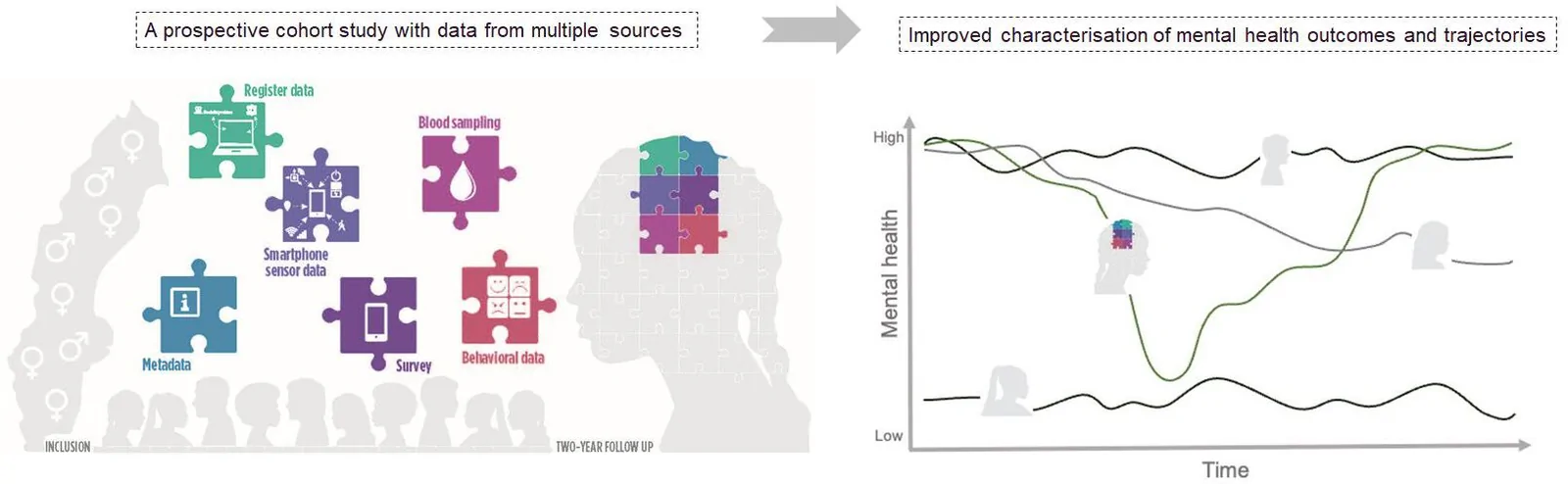
Illustration of how data from multiple sources will be used to improve characterisation of mental health outcomes and trajectories over time.

As outlined in figure 2, three different data collection protocols will be tested for their feasibility and effectiveness in characterising and predicting mental health outcomes. After the baseline assessment, participants are assigned to protocols within the app using stratified random allocation to ensure balanced representation of age and sex.

**Figure 2:**
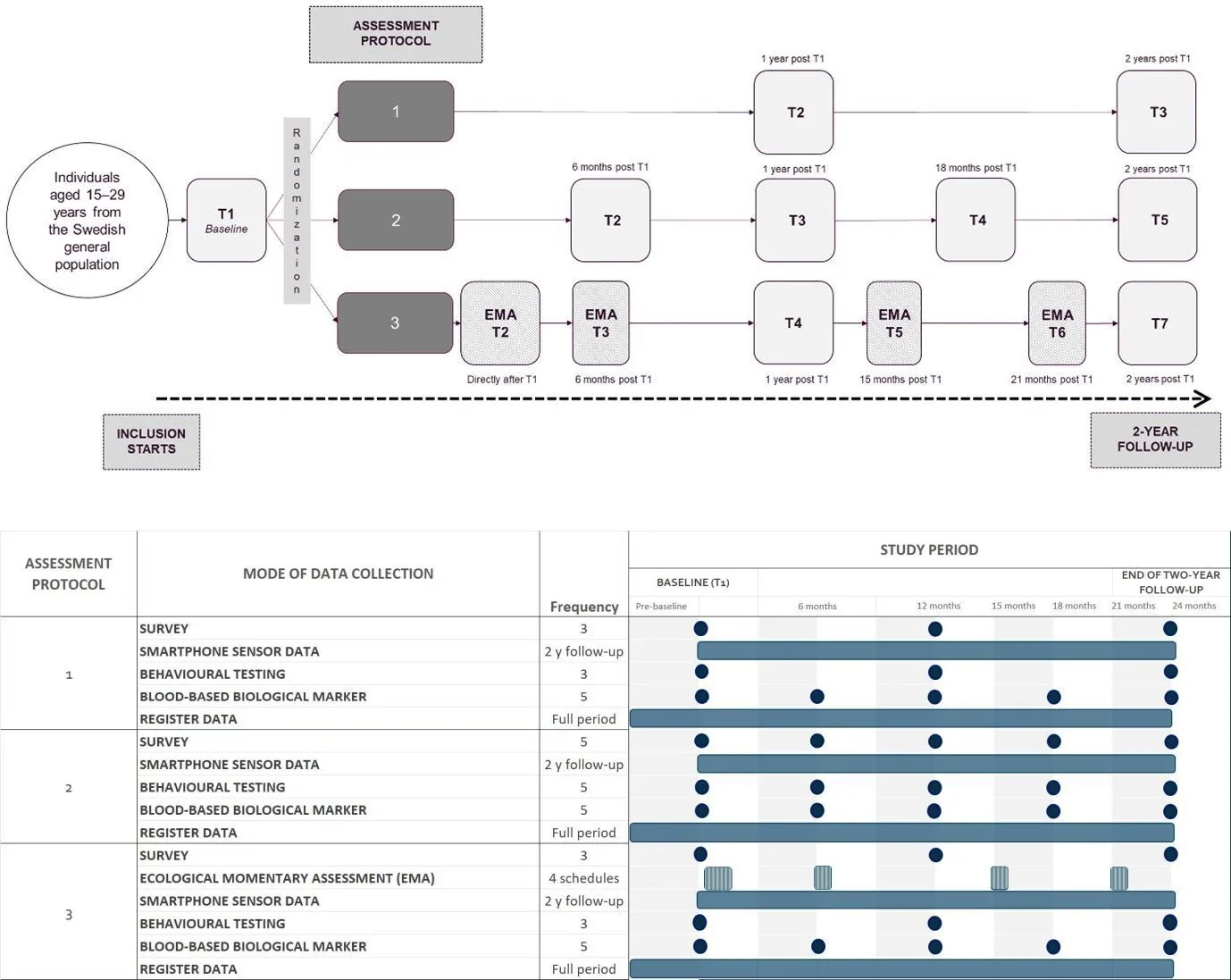
(A,B). Overview of the data collection schedules, participant allocation and assessment timelines across the three study protocols throughout the 2-year study period. EMA, ecological momentary assessment.

In *Protocol 1*, data are collected on three occasions: at baseline and follow-ups at 1 and 2 years after baseline. This design with yearly assessments is congruent with conventional methods of assessment by surveys.^eg,35^ To investigate the potential benefits as well as risks of more frequent assessments, data are collected in the same way in *Protocol 2*, but on five occasions: at baseline and 6, 12, 18 and 24 months later. In *Protocol 3*, the yearly assessments (T1, T4, T7) are complemented by EMA. After the baseline assessment, participants assigned to *Protocol 3* are asked to complete daily registrations using a selection of self-report measures during a 30-day period (first EMA period: T2) and subsequently for a 14-day period (EMA period 2–4: T3, T5, T6) (figure 2). The EMA schedule is repeated at 6 months from baseline (year 1), 15 months from baseline (year 2), and 21 months from baseline (year 2) to control for seasonal variation in mental health and engagement in health behaviours.

Participants receive incentives upon completing assessments. A 100 SEK gift card (~US$11) is awarded for each completed assessment, that is after finishing a survey, completing behavioural tests and/or providing a blood sample. Consequently, participants may receive between 100 and 300 SEK per assessment occasion, depending on the number of assessment activities completed. Further, participants receive an additional 100 SEK gift card upon completing the EMA schedule.

#### Survey

Participants complete a range of surveys via the UPIC app using established self-report measures. These surveys capture information of mental ill-health and mental well-being, as well as psychological constructs (eg, resilience, emotion regulation, psychological flexibility, curiosity), health behaviours, social relationships, sociodemographic and health characteristics.

The primary *mental ill-health* outcomes are symptoms of depression and anxiety. Symptoms of depression are measured with the Swedish version of the Patient Health Questionnaire—9 (PHQ-9+1),^59^ and symptoms of anxiety with the linguistically validated Swedish version of the Generalised Anxiety Disorder 7-item scale (GAD-7),^60^ both provided on the Patient Health Questionnaire Screeners website.^61^ Secondary outcomes of mental ill-health include perceived stress (Perceived Stress Scale-10, PSS-10),^62 63^ sleep disturbances (Insomnia Severity Index, ISI).^64 65^ The primary outcomes of *mental well-being* are assessed using the WHO—Five Well-being Index (WHO-5)^66 67^ and the Mental health short continuum—Short Form (MHC-SF).^68 69^ For measures lacking validation in Swedish adolescent and young adult populations, psychometric properties will be evaluated and reported as appropriate. For instance, psychometric properties of the GAD-7 have recently been evaluated in Swedish AYAs.^70^

*Psychological construct*s are assessed using the Adolescents’ Emotion Regulation Strategies Questionnaire–Extended,^71^ a short version (12 items) of the Multidimensional Psychological Flexibility Inventory,^72 73^ a short version (7 items) of the Five-Dimensional Curiosity Scale Revised^74^ and the Resilience Scale.^75 76^ *Sociodemographic and health characteristics*, including country of birth, gender identity, age, education, living conditions, social relationships, physical health, earlier adversities and significant life events (adapted Stressful Life Events Scale)^77^ and experience of violence and harassment are assessed using adapted questions from Swedish national health surveys.^4^ *Health behaviours* (eg, physical activity, alcohol and drug use, social media use) are assessed using questions from Swedish public health surveys,^4^ the National Board of Health and Welfare and the Swedish Media Council.^78^

Participants allocated to the third assessment protocol will complete EMA in addition to annual surveys. EMA self-report questions are delivered via the UPIC app in sets of 25 items per session (period 1), and 16 items per session (periods 2–4). These items are drawn from validated measures of mental ill-health, well-being and engagement in health-related behaviours.

#### Behavioural tests

Data from online, remote behavioural tests are used to assess basic psychological mechanisms of importance for mental health, including avoidance behaviour, executive functions and decision-making under risk and ambiguity. Participants are linked from the UPIC app to a secure website hosted at Uppsala University to perform the following tests:

- *Approach-Avoidance Conflict Task*: In a series of trials, participants choose between viewing aversive images+sounds and get a reward (points) or neutral images+sounds for no reward.^79^ This induces an incentive-conflict between avoiding the aversive stimulus and attaining the reward and is used here as a measure of costly avoidance (ie, avoidance that comes at a cost).
- *ECAN Card Sorting Test*: Participants are tasked with sorting 64 cards according to non-disclosed predefined rules (colour, symbol or number) that change after every 10 cards in a row correctly sorted. This test assesses cognitive flexibility (set-shifting), how fast the participants adapt to a new strategy when the rules suddenly change. This is an online adaptation of the Wisconsin Card Sorting Test 64 Card version using a stop rule of 3 correctly sorted categories or all 64 cards.^80 81^
- *Block Tapping*: A visuospatial short-term memory task where participants in a series of trials are presented with blocks that change colour in a specified order and asked to repeat each sequence immediately after presentation. This is a variation of the Corsi Block Tapping Test with the layout of the 10 blocks changing within a fixed larger matrix for each trial.^82 83^
- *Wheel of Fortune:* This gambling task consists of a number of risky and ambiguous trials where participants choose between two wheels-of-fortune, one is always safe (certain to win five points) and the other a gambling option which could yield higher reward but also no reward.^84 85^ The higher reward varies across trials between 5, 8, 20 and 50 points and the probability to win varies between 0.125, 0.25, 0.375, 0.5, 0.625 and 0.75. The gambling option is presented as a circle with blue and red proportions indicating the probability of winning (blue) or losing (red). The safe option is a blue circle indicating the certain win. In risky trials, the gambling option shows the probability of winning, whereas in the ambiguous trials, the gambling option circle is largely covered by a grey part obscuring the full probability of winning and introducing ambiguity. Ambiguity levels vary between 25%, 50%, 75% and 100%. Hence, the task assesses decision-making under risk (known probability) and ambiguity (unknown probability) and provides measures of risk and ambiguity attitudes.

Participants’ choices, correct answers, response/reaction time and response pattern (including button clicks) are collected.

#### At-home blood tests

A random subset (n=200) is asked to provide blood using easy and successful remote sampling kits where blood is collected from a finger prick (two microsamplers of 30 µL blood specimen, https://www.neoteryx.com/home-blood-blood-collection-kits-dried-capillary-blood). When the samples have dried the participants will send back the samples in a prepaid envelope for analysis. Biological markers are extracted from the Neoteryx VAMP tips and analysed with liquid chromatography and high-resolution tandem mass spectrometry after addition of stable isotope-labelled standards for absolute quantification. This enables precise monitoring of about 1800 biomarkers and ratios of those, carefully selected to target the most important metabolic pathways, including steroid-, hormone-, neurotransmitter- and lipid regulation. The analysis will provide test results in ng/mL for each substance.

#### Passive data from smartphone sensors

Passive data are continuously collected via the UPIC app and serve as proxies for behavioural patterns, including phone usage, physical activity, sleep and mobility. The feature modalities include:

- *Data usage* including internet activity (kb/s). The UPIC app records accumulated upload and download rates together with timestamps, providing an indicator of overall internet activity. In addition, reachability is captured through timestamped smartphone connectivity status, indicating whether the device is connected to a cellular network, Wi-Fi or neither.
- *General smartphone usage:* Data are collected on screen activity, charging status and device reboot.
- *Geographical movement patterns* are measured with location services (GPS). The actual GPS location is not recorded, but only by a random offset. Geographical movement patterns are furthermore measured by raw accelerator data, collected when the motion exceeds a certain threshold; motion activities, including being stationary, walking, running, cycling and movement in a vehicle, are recorded when the state changes. For iOS, we collect device motion to provide more detailed motion sensor data.

#### App metadata

The use of app-based surveys enables the collection of behavioural metadata alongside self-reported responses. Such metadata may provide clinically relevant insights into cognitive processes, including attention, processing speed and working memory capacity, as well as potential deterioration in psychiatric symptoms. Specifically, the time at which a survey is opened, the duration taken to answer each question and complete the survey, and any changes made to responses during completion are recorded.

#### National health registries

The following register data are linked to obtain retrospective (prebaseline) and prospective data on mental health, sociodemographic and labour market participation:

- *The National Patient Register,* held by the National Board of Health and Welfare, is used to obtain information on psychiatric diagnosis and psychiatric outpatient and inpatient care including date of admission and length of stay (based on F-codes ‘Mental and behavioural disorders’ according to International Classification of Diseases, Tenth Revision (ICD-10)).
- *The National Prescribed Drug Register*, held by the National Board of Health and Welfare, is used to obtain information on the redeeming of psychotropic medication according to yearly prescriptions of anxiolytics, sedatives/hypnotics and antidepressants (Anatomical Therapeutic Chemical (ATC) codes; N05B, N05C and N06A).
- *The LISA (Longitudinal integrated database for health insurance and labour market studies) and Population by Labour market status (BAS) register*, held by Statistics Sweden, provide data on civil status, country of birth, educational attainment, income, work status, including occupational class and employment sector and sickness absence (duration and cause/diagnosis).

### Preliminary data analysis plan

The study has been registered in ClinicalTrials.gov (UU20230127; NCT: NCT06490120) with the aim to establish a comprehensive, population-based dataset to examine mental health trajectories among AYAs (15–29 years) in Sweden over a 2-year period. This dataset will combine data from multiple sources for comprehensive identification, characterisation and profiling of mental health outcomes and trajectories.

Data will be analysed to advance understanding of mental health among AYAs, including both mental ill-health and well-being outcomes. The analytical strategy is designed to characterise mental health outcomes and their determinants, including age-based prevalence estimates, model trajectories over time, examine associations with demographic, psychosocial, behavioural and biological factors, evaluate the added value of integrating data from multiple sources and assess feasibility and acceptability of study procedures.

To model mental health trajectories and identify meaningful subgroups, data-driven techniques such as growth mixture and trajectory modelling, latent class analysis, network analysis and clustering of behavioural and digital phenotyping data will be applied. These methods will support identification of distinct trajectories, risk profiles and behavioural patterns across the 2-year follow-up, with the possibility to include both predefined patterns across the three main assessments and fully data-driven trajectory solutions capturing intra-individual change over time.

Analyses will also evaluate whether integrating multiple data sources—surveys, behavioural tests, smartphone sensor data, registry data and biomarkers—improves prediction compared with models using data from a single source. Models will also compare predictive utility and temporal sensitivity across the three assessment protocols. Predictive modelling will draw on traditional machine learning (eg, random forests, gradient boosting, penalised regression) and deep learning methods. Because the data streams from the different data sources vary in scale, structure and format, harmonisation of formats, terminologies and measurement units is essential before predictive modelling.^86 87^ The multi-source data collection relative to the sample size will be carefully considered in the analyses to minimise the risk of overfitting and ensure robust findings. Missing data will be assessed and reported for all data sources. For surveys, missing data will be imputed following established guidelines for each measure, including imputation where appropriate. Missingness in data from other sources will be addressed using approaches suitable for the specific data structure and analytical method employed, and the handling of missing data will be reported in detail for each analysis.

Feasibility will be evaluated through recruitment rates, demographic representativeness, retention and attrition patterns across the three assessment protocols, completion rates for surveys, behavioural tests, EMA prompts and sensor data. These indicators will be summarised descriptively, evaluated in relation to predefined feasibility targets and benchmarks established prior to analysis, and compared across the three assessment protocols.

Quantitative indicators will be complemented by descriptive assessments of technical performance, including data completeness across data sources. Acceptability will be assessed using participant-reported indicators of usability, burden and engagement, gathered through app-based usage logs. In addition, analyses will examine factors associated with variability in feasibility outcomes, including predictors of low compliance and reduced follow-up participation.

### Patient and public involvement

Representatives from the target population (AYAs aged 15–29 years) have been actively involved in the UPIC programme since its launch in 2023. Two AYAs were engaged from the outset as research partners for the current study, participating in research team meetings and contributing to key decisions. One was recruited from the nation-wide, non-profit organisation ‘Mind’ working to promote mental health and the other by a call-out among students at Uppsala University. In addition, AYAs were recruited to a reference group that provided input throughout the study development process. Group members participated in specific tasks, such as design thinking, pilot testing of the app used for data collection and refining recruitment strategies and study materials to ensure inclusivity across gender identities and cultural backgrounds. To facilitate broad participation from young people across Sweden, five rounds of online workshops were conducted. In each round, separate workshops were held for two age groups (15–20 and 21–29 years), resulting in a total of 10 workshop groups with approximately 2–5 participants per group. The workshops provided opportunities for AYAs from different regions to share perspectives and provide feedback on study procedures.

To date, the reference group co-created the study-specific mobile application (the UPIC app)—including its features and layout—and has contributed to the evaluation of surveys and the development of participant information materials. They were also consulted on the burden of participation and time requirements during pilot testing. Moving forward, research partners will be involved in data interpretation, dissemination of findings and development of future interventions. This collaborative approach ensures that the study remains relevant, acceptable and informed by the priorities and experiences of the target population.

## Ethics and dissemination

This study has been approved by Swedish Ethical Review Authority (2023–00246-01, 2023–05652-02, 2024–02953-02). Strict security procedures are in place, including emergency procedures in the unlikely event of a data breach. All data processing complies with the General Data Protection Regulation (GDPR), including pseudonymisation, secure storage and protection of personal information. Participants are fully informed about the aims of the study, confidentiality measures and data security procedures. Informed consent is obtained at registration to the study in the UPIC app, with participants able to provide consent for each data collection source. They are informed that participation is voluntary and can be withdrawn at any time without providing a reason.

The use of a digital mobile application is not expected to lead to any direct risks to the participants’ health. However, some participants may experience distress when responding to mental health questions. To support this, participants are encouraged to contact the UPIC team for further guidance via email. Participants reporting more severe symptoms of depression (≥14 of the PHQ-9)^88^ or who report having had suicidal thoughts/attempts within the past 12 months will receive automated messages through the UPIC app with information on available help and support services. Risks from blood sampling are minimal and we have experience distributing relevant information on how the tests are performed, managed and used.

Research findings will be disseminated through international peer-reviewed journals and presented at scientific conferences, the UPIC website and to user and advocacy groups.

### Strengths and limitations

This study focuses on individuals in a critical developmental window for mental health and health-behaviour formation. By recruiting participants aged 15–29 years, we capture key transitions from compulsory schooling to upper secondary education, higher education and entry into the labour market. Establishing a comprehensive dataset for this age group is pivotal for guiding precision prevention and intervention strategies.

A key strength is the study’s design, combining data from multiple sources and integrating behavioural, social, psychological and biological dimensions of mental health. This approach enables detailed characterisation of mental health outcomes and trajectories and the identification of risk and protective factors across different domains. An additional strength is the randomisation to three data assessment protocols, enabling efficient evaluation of their relative effectiveness, feasibility and participant burden to identify the most sustainable and informative approaches for future large-scale studies. Yet, non-participation and attrition represent important challenges, particularly for building a representative dataset that adequately reflects the diversity of young people’s experiences. To reduce the risk of selection bias, random sampling is complemented with targeted recruitment efforts aimed at under-represented groups. Register-based information on education, residential area and country of birth will be used to evaluate the distribution and composition of the recruited cohort. These variables are derived from Swedish national registers, which maintain high accuracy and population coverage through the use of personal identity numbers, enabling precise linkage between datasets.

To support retention, participants receive incentives in the form of gift cards. Linkage to health registers will also partly compensate for missing self-reported outcomes by providing objective information on diagnoses, prescriptions and healthcare utilisation. While the study benefits from the use of multiple data sources, the strong reliance on self-reported measures remains a notable limitation, introducing potential sources of reporting bias arising from misinterpretation of items or socially desirable responses. In addition, although the study incorporates behavioural and register-based data, information from parents, peers, teachers, partners or other significant individuals is not included, which represents a potential area for future follow-up waves and add-on studies. Consequently, the study primarily captures mental health from the participants’ perspective and may not fully reflect broader social and contextual perspectives. Nonetheless, the combination of survey data with behavioural tests, digital phenotyping and high-quality register data substantially strengthens the validity, robustness and interpretability of the overall dataset.

To conclude, mental health among AYAs represents a growing public health challenge, reflected in rising rates of mental ill-health across Sweden and internationally. This study responds to these trends by establishing the first Swedish cohort to combine biological, behavioural, digital phenotyping and national register data to characterise mental health among AYAs in real-world contexts. This multidimensional, longitudinal approach provides an unprecedented opportunity to map mental health trajectories, understand their determinants and examine patterns of risk and resilience during a critical developmental period.

By integrating repeated surveys, behavioural tests, passive smartphone data, EMA, biomarkers and register linkages, the study will generate high-resolution, temporally rich data capable of capturing the dynamic interplay between biological, psychological, social and behavioural factors. Evaluating the feasibility and acceptability of these diverse data collection strategies, including participant engagement over time, will inform scalable, sustainable models for future youth mental health surveillance and cohort studies.

Generating a comprehensive, population-based dataset provides the scientific foundation needed for effective, evidence-based preventive strategies. The resulting dataset will support exploratory analyses and predictive modelling using both traditional statistical methods and advanced machine learning, enabling the identification of the most informative data sources and combinations of data. In public health, normative data serve as essential benchmarks for assessment and policy development, yet comparable standards for mental health among AYAs are currently lacking. Establishing such a dataset is therefore crucial. Moreover, this cohort can be considered a pilot for the development of a broader research infrastructure in this field, and if it proves feasible for larger-scale implementation, it can form a core component of a national research infrastructure for youth mental health, supporting future public health monitoring.

Together, these efforts will build a much-needed empirical foundation for precision mental health research, informing timely, personalised preventive interventions and strengthening life-course approaches to mental health among AYAs.
