# Supplementary material for: The ‘UPIC’ cohort: a nationwide prospective study of mental health among adolescents and young adults in Sweden – a study protocol
Source: BMJ Open. 2026 Sep 21;16(9):e120432. doi: 10.1136/bmjopen-2026-120432 (PMC13630165; doi:10.1136/bmjopen-2026-120432)
Supplement: Supplementary file 1 [file bmjopen-2026-120432.reviewer_comments.pdf]

PEER REVIEW HISTORY

BMJ Open publishes all reviews undertaken for accepted manuscripts. Reviewers are asked to complete a checklist review form and are provided with free text boxes to elaborate on their assessment. These free text comments are reproduced below.

ARTICLE DETAILS

Title (Provisional)

THE "UPIC" COHORT: A NATIONWIDE PROSPECTIVE STUDY OF MENTAL HEALTH AMONG ADOLESCENTS AND YOUNG ADULTS IN SWEDEN - A STUDY PROTOCOL

Authors

Hovén, Emma; Frick, Andreas; Skalkidou, Alkistis; Papadopoulos, Fotios C; Ekselius, Lisa; Gingnell, Malin; Karlsson, Rose-Marie; Brocki, Karin; Ljungvall, Hanna; Lindqvist, Emma Ahlqvist; Eklund, Rakel; Bergquist, Jonas; Eklund, Lina; Nilses, Ellen; Hallsén, Stina; Åsenlöf, Pernilla

---

VERSION 1 - REVIEW

**Reviewer**	**1**
**Name**	**Giannotta, Fabrizia**
**Affiliation**	**University of Turin**
**Date**	**16-Jul-2026**
**COI**	**None**

The paper presents a protocol for a very ambitious and important project that aims to integrate multi-source data to explain the rise in psychological problems among youth and young adults. The project is both ambitious and highly relevant. While the methodological and ethical sections are very clear, I have a few comments regarding the rationale for the project and its theoretical background.

Specifically, the authors focus on the need to identify factors associated with poor mental health, citing a general lack of research in this area. This claim is not entirely accurate: a substantial body of work on the determinants of mental health has been published across disciplines—from psychology and psychiatry to public health—and particularly in Sweden (the authors cite a 2021 FORTE report, but many further studies have appeared since then), but more importantly, it is not clear which specific factors will be targeted by the many instruments the authors intend to use. Without a theoretical framework to guide the selection of these multiple factors across different contexts, the choices appear arbitrary and insufficiently justified. A clearer theoretical rationale would help the reader appreciate the

considerable effort involved in collecting so much data through so many different instruments.

**Reviewer**	**2**
**Name**	**Eriksson, Charli**
**Affiliation**	**Karolinska Institutet**
**Date**	**24-Jul-2026**
**COI**	**None**

Review: The ”UPIC” Cohort, BMJ Open 2026

It is an impressive study that is described in the protocol, which is in line with the guidelines of BMJ Open. It is important to establish a comprehensive dataset combining data from multiple sources (surveys, behavioural tests, registers and smartphone sensors) to assess mental health, including mental ill-health and well-being. It can result in important new understanding of the development of mental health among adolescents and young adults (AYAs).

Major concern:

There are conceptual ambiguities in the use of concepts like “poor mental health”, “mental ill-health” and “mental well-being”. Are you subscribing to a one-dimensional mental health as you on page 5 write “Mental ill-health is commonly conceptualized a continuum ranging from common symptoms of distress – such as worry, stress, and emotional strain – to severe mental disorders, including major depressive disorder and psychosis”? It may be appropriate to include some lines on the dual-dimension (or factor) model, which emphasises the need for comprehensive mapping om mental health.

Another concern is that the time span for the study is just two years, which is never discussed or giving good argument for.

The use of the term “multiple sources” refers to different data collection methods. It is still from just the selected persons. No information is collected from parent, teachers/workers, peers or other significant persons in the life of the participants. In this sense the study has an individual focus not considering the complexity of mental health.

Minor concerns

Page 6, line 17: “In Sweden, nationally representative studies providing comprehensive estimates of common mental health conditions, such as depression and anxiety, remain scarce”. You refer to a report in the grey literature from 2021. There are now several such studies using surveys as well as national registry data.

Page 6, line 24: Is reference 22 the right reference to use here?

Page 6, line 43: Are all those outcomes covered in the references? You do not mention cannabis use which is the focus in one of the references.

Page 7, line 59: It would be of interest for the reader to be informed about compliance in earlier studies using this research strategy.

Page 10, line 15: Why use two different random subsamples? Please give arguments for this procedure.

Page 10, line 36: It is interesting to note that the initial sample was shew towards female participants. How well did you manage to adjust for that in the subsequent actions? Maybe this is a result that should be presented in subsequent papers, but it could be added here.

Page 11, line 36: Figure 2 is very illustrative for complex data collection. Maybe the months should be included.

Page 12, line 22. Maybe the range of money that the participants can achieve would be informative.

Page 12, line 49 -: When referring to the different scales used, you refer to the original publications on the scales: It could be informative to also get references to the Swedish translation and validation of the scales if such papers are available. If not, do you plan to undertake a psychometric analysis of the scales? This applies to all scales used in the data collection. No mention of how to deal with multiple information from rather few participants. The method for the random selection is described.

Page 13, line 31: which data is coming from the NBHW?

Page 14, line 15 and following. No references to the three other behavioural tests.

Page 15, line 17: The at-home blood test is poorly described. There is no specific research question nor any arguments or references to literature.

Page 18, line 38: the data analysis is not presented in many details. The method of imputation needs to be presented.

Page 18, line 47: This is a concise description of a key aspect in the study. It is not clear how inferences will be drawn using the different indicators.

Page 19, line 26: How many different reference groups were used? Should also the participation in the online workshops be presented?

Page 20, line 45: The ethical aspects are well covered.

Page 23, references: The literature is mostly well covered. The list of literature should include English translation of the titles within brackets. This should be added in references number 4, 6 and 57. Reference no 21 and 57 are incomplete.

VERSION 1 - AUTHOR RESPONSE

REVIEWER: 1
DR. FABRIZIA GIANNOTTA, UNIVERSITY OF TURIN

<u>Comments to the Author:</u>
The paper presents a protocol for a very ambitious and important project that aims to integrate multi-source data to explain the rise in psychological problems among youth and young adults. The project is both ambitious and highly relevant. While the methodological and ethical sections are very clear, I have a few comments regarding the rationale for the project and its theoretical background.

Specifically, the authors focus on the need to identify factors associated with poor mental health, citing a general lack of research in this area. This claim is not entirely accurate: a substantial body of work on the determinants of mental health has been published across disciplines—from psychology and psychiatry to public health—and particularly in Sweden (the authors cite a 2021 FORTE report, but many further studies have appeared since then), but more importantly, it is not clear which specific factors will be targeted by the many instruments the authors intend to use. Without a theoretical framework to guide the selection of these multiple factors across different contexts, the choices appear arbitrary and insufficiently justified. A clearer theoretical rationale would help the reader appreciate the considerable effort involved in collecting so much data through so many different instruments.
<u>Authors' response:</u>
We thank the reviewer for this important comment. We agree that a substantial body of research has examined determinants of mental health across disciplines, and we acknowledge that the evidence base has continued to grow since the publication of the 2021 FORTE report. We have revised the manuscript accordingly and included additional literature to better reflect the current evidence base.t. Our intention was not to suggest a lack of research on determinants per se, but rather to emphasize the need for studies that integrate diverse theoretical perspectives and methodological approaches to address the complexity of mental health among AYAs.

We also appreciate the request for a clearer rationale regarding the selection of factors included in the study. The choice was informed by existing literature available at the time of study conception and developed through extensive interdisciplinary discussions within the UPIC research programme. UPIC works within a biopsychosocial framework, bringing together researchers from psychology, social sciences, epidemiology, behavioural medicine, and related fields, providing complementary perspectives on mental health and its determinants. Against this background, an important rationale for establishing the cohort was to create a rich dataset that enables UPIC researchers from different disciplines to address emerging research questions, test and generate hypotheses, and apply theoretically informed models. In addition, within the interdisciplinary environment of UPIC, the present cohort study is complemented by related studies exploring how mental health and mental ill-health are conceptualised, communicated, and experienced by young people.

Consistent with the biopsychosocial framework underpinning the study, the measures were selected to capture psychological, social, physical, behavioural, biological, and health-related dimensions that have been identified as relevant in previous research. This approach aligns with the overarching aim of examining mental health as a complex phenomenon emerging through interactions among multiple levels of influence.

To clarify this rationale, we have expanded both the Introduction and Methods sections to more clearly describe the theoretical framework underpinning the study and its role in guiding the selection of measures (see revised manuscript, pages 6-7 and 9-10).

REVIEWER: 2
DR. CHARLI ERIKSSON, KAROLINSKA INSTITUTET

<u>Comments to the Author:</u>
Review: The ”UPIC” Cohort, BMJ Open 2026
It is an impressive study that is described in the protocol, which is in line with the guidelines of BMJ Open. It is important to establish a comprehensive dataset combining data from multiple sources (surveys, behavioural tests, registers and smartphone sensors) to assess mental health, including mental ill-health and well-being. It can result in important new understanding of the development of mental health among adolescents and young adults (AYAs).
<u>Authors' response:</u> We thank the reviewer for these encouraging remarks and for the thorough and constructive review of our manuscript. We greatly appreciate the valuable comments and suggestions, which we believe have helped us to improve the manuscript substantially.

Major concern:
There are conceptual ambiguities in the use of concepts like "poor mental health", "mental ill-health" and "mental well-being". Are you subscribing to a one-dimensional mental health as you on page 5 write "Mental ill-health is commonly conceptualized a continuum ranging from common symptoms of distress – such as worry, stress, and emotional strain – to severe mental disorders, including major depressive disorder and psychosis"? It may be appropriate to include some lines on the dual-dimension (or factor) model, which emphasises the need for comprehensive mapping om mental health.
<u>Authors' response:</u> Thank you for this important comment. We agree that the original version of the manuscript did not sufficiently describe the conceptualization of mental health and use of different terms for mental ill-health and mental well-being. The design of the study and the selected outcome measures are informed by the dual-factor (dual-dimension) model of mental health, which emphasizes the importance of comprehensively assessing both dimensions of mental health. We have therefore revised the Introduction to better describe this conceptual framework and its relevance to the study (page 6).

Another concern is that the time span for the study is just two years, which is never

discussed or giving good argument for.
<u>Authors' response:</u> The rationale for the two-year follow-up relates to the central aim of the present study: to evaluate whether an intensive, multi-source, longitudinal approach to studying AYAs is feasible, acceptable, and sustainable. Given the substantial resources and costs associated with collecting repeated, in-depth data across multiple domains, it was considered important to first establish and evaluate the feasibility and utility of this approach before deciding on the final design, sample schedule, measures and committing to a longer follow-up period.

At the same time, the data collected over this period are expected to provide an important contribution to understanding fluctuations and patterns in mental health among AYAs, while also generating hypotheses for future follow-up studies. While a longer follow-up would provide additional opportunities to study mental health trajectories, the selected timeframe is expected to capture meaningful changes in mental health and provide a strong foundation for future follow-up studies and further development of the cohort. Thus, the two-year period represents a balanced and pragmatic first step that both evaluates the feasibility of this resource-intensive study design and generates important scientific data, while also establishing a foundation for potential future follow-up studies and further development of the cohort. The rationale for the two-year follow-up has been clarified in the revised manuscript (pages 10-11).

The use of the term "multiple sources" refers to different data collection methods. It is still from just the selected persons. No information is collected from parent, teachers/workers, peers or other significant persons in the life of the participants. In this sense the study has an individual focus not considering the complexity of mental health.
<u>Authors' response:</u> We thank the reviewer for this important comment. We use the term "multiple sources" to refer to the integration of different types of data and data collection methods. We agree that incorporating information from significant others, such as parents, peers, teachers, or partners, could provide valuable complementary perspectives on mental health and contribute to a more comprehensive understanding of mental health trajectories over time, as well as the role of social relationships and broader social and societal contexts in shaping mental health.

While the inclusion of such perspectives would have been valuable, it was beyond the scope of the current study. The primary aim was to evaluate the feasibility and utility of a comprehensive, multi-method longitudinal approach in a large population-based sample of adolescents and young adults. Nevertheless, the reviewer raises an important point, and the inclusion of informant data from significant others represents a promising direction for future research building on the UPIC cohort. To acknowledge this, we have added a statement to the revised manuscript (page 24).

Minor concerns
Page 6, line 17: "In Sweden, nationally representative studies providing comprehensive estimates of common mental health conditions, such as depression and anxiety, remain scarce". You refer to a report in the grey literature from 2021. There are now several such studies using surveys as well as national registry data.

<u>Authors' response:</u> We have revised this sentence accordingly and the reference list has been updated.

Page 6, line 24: Is reference 22 the right reference to use here?
<u>Authors' response:</u> We thank the reviewer for this observation. Reference 22 was intended to support the need for multidimensional assessment approaches that capture the complexity and heterogeneity of mental health. To improve precision, we have revised the wording to better reflect the scope of the cited work.

Page 6, line 43: Are all those outcomes covered in the references? You do not mention cannabis use which is the focus in one of the references.
<u>Authors' response:</u> To better align the text with the cited literature, we have revised the sentence to include cannabis use among the listed health behaviours associated with mental health outcomes (page 7).

Page 7, line 59: It would be of interest for the reader to be informed about compliance in earlier studies using this research strategy.
<u>Authors' response:</u> We thank the reviewer for this suggestion. We have added information on compliance from previous studies in the revised manuscript (page 8).

Page 10, line 15: Why use two different random subsamples? Please give arguments for this procedure.
<u>Authors' response:</u> The two random subsamples were invited sequentially because the recruitment rate was uncertain at the outset. This approach allowed us to evaluate recruitment after the first invitation wave before deciding whether additional invitations were needed, thereby reducing the risk of recruiting more participants than could be accommodated within the study and avoiding unnecessary postal costs. We have clarified this rationale in the manuscript (page 11).

Page 10, line 36: It is interesting to note that the initial sample was shew towards female participants. How well did you manage to adjust for that in the subsequent actions? Maybe this is a result that should be presented in subsequent papers, but it could be added here.
<u>Authors' response:</u> We agree that the initial overrepresentation of female participants and the effectiveness of subsequent recruitment adjustments are important feasibility outcomes. Preliminary analyses indicate that the initial gender distribution, with approximately 75% female and 25% male participants, shifted to approximately 60% female and 40% male following the implementation of targeted recruitment strategies. As these analyses are part of the planned feasibility outcomes, we have not added these preliminary results to the manuscript. The impact of the recruitment strategy, as well as broader issues related to sample representativeness, will be examined and reported in future publications focusing on recruitment, representativeness, and feasibility.

Page 11, line 36: Figure 2 is very illustrative for complex data collection. Maybe the months should be included.
<u>Authors' response:</u> Revised accordingly.

Page 12, line 22. Maybe the range of money that the participants can achieve would be

informative.
<u>Authors' response:</u> **Thank you for this suggestion. We have revised the manuscript to clarify the compensation provided to participants. Specifically, we now state that participants may receive between 100 and 300 SEK per assessment occasion.**

Page 12, line 49 -: When referring to the different scales used, you refer to the original publications on the scales: It could be informative to also get references to the Swedish translation and validation of the scales if such papers are available. If not, do you plan to undertake a psychometric analysis of the scales? This applies to all scales used in the data collection. No mention of how to deal with multiple information from rather few participants. The method for the random selection is described.
<u>Authors' response:</u> **The reviewer raises an important point. Where available, we have added references to Swedish translations and validation studies of the measures used in the study. For measures lacking Swedish validation studies in adolescent and young adult populations, psychometric properties will be evaluated and reported as appropriate within the UPIC programme. For example, as a first step, we have recently published a psychometric evaluation of one of the primary outcome measures, the GAD-7, for this sample (https://doi.org/10.1186/s12888-026-08423-0), and this reference has now been added to the manuscript. In addition, a psychometric evaluation of the PHQ-9 has been completed and the manuscript is under review. In the revised manuscript, we also acknowledge the challenge of analysing extensive multi-source data from a finite sample size (page 20).**

Page 13, line 31: which data is coming from the NBHW?
<u>Authors' response:</u> **Thank you for this comment. The health behaviour items were selected after reviewing well-established questions from Swedish public health surveys, surveys from the National Board of Health and Welfare (NBHW), and the Swedish Media Council. Specifically, from a NBHW survey on living conditions, we included questions assessing breakfast consumption and alcohol use (number of standard drinks per week and frequency of consuming four or more standard drinks on the same occasion). However, because the health behaviour battery was developed using questions from multiple established sources and several items were adapted for the present study, we chose not to specify the source of each individual question in the manuscript in the interest of brevity and readability.**

Page 14, line 15 and following. No references to the three other behavioural tests.
<u>Authors' response:</u> **Thank you for this observation. We have revised the manuscript and completed the description of the behavioural tests by adding the relevant references for the three tests that were previously lacking citations (new references for the behavioural tests: 79-84)**.

Page 15, line 17: The at-home blood test is poorly described. There is no specific research question nor any arguments or references to literature.
<u>Authors' response:</u> **In the revised manuscript, we have clarified the scientific rationale for including biological markers assessed through at-home blood sampling. Specifically, we now state more clearly that the study is guided by an interdisciplinary framework in which mental health is understood as the result of complex interactions among psychological, social, behavioural, and biological factors (page 7 and 9).**

Within this framework, the integration of biological data will be examined to explore whether it has the potential to improve the characterization of mental health outcomes. Given the exploratory nature of this component, the analyses are primarily hypothesis-generating and intended to inform future studies examining the role of biological factors in mental health outcomes.

In addition, the at-home blood sampling component serves an important feasibility purpose by evaluating the acceptability and utility of collecting biological data in a population-based cohort of AYAs.

Page 18, line 38: the data analysis is not presented in many details. The method of imputation needs to be presented.
<u>Authors' response:</u> Given the broad range of outcomes and data sources included in the study, no single imputation method is appropriate for all analyses or measures. We have therefore clarified that missing survey data will be handled according to established recommendations for each measure, while missing data in other modalities (e.g., sensor, registry, biomarker, and behavioural test data) will be addressed using methods appropriate to the respective data structure and analysis. The specific approach used will be reported for each analysis in forthcoming papers.

Page 18, line 47: This is a concise description of a key aspect in the study. It is not clear how inferences will be drawn using the different indicators.
<u>Authors' response:</u> We have clarified how inferences will be drawn from the different indicators by specifying that feasibility indicators will be summarized descriptively, evaluated in relation to predefined feasibility targets and benchmarks established prior to analysis (work in progress), and compared across the three assessment protocols. We have also clarified that the findings will be used to assess the feasibility of the study procedures and inform future large-scale implementation.

Page 19, line 26: How many different reference groups were used? Should also the participation in the online workshops be presented?
<u>Authors' response:</u> We agree that this needed clarification. We have revised the text to clarify that AYAs were involved through a single reference group that contributed to different study activities and assignments throughout the study development process. We have also added information about the online workshops, see pages 20-21. A more detailed description of the reference group involvement and co-creation process is provided in a separate manuscript that is currently under review.

Page 20, line 45: The ethical aspects are well covered.

Page 23, references: The literature is mostly well covered. The list of literature should include English translation of the titles within brackets. This should be added in references number 4,
6 and 57. Reference no 21 and 57 are incomplete.
<u>Authors' response:</u> Thank you for this observation. We have updated the reference list by adding recent and relevant references to ensure that the literature base is comprehensive. In addition, we have reviewed the reference list for accuracy and completeness.
